# Supplementary material for: Two major ecological shifts shaped 60 million years of ungulate faunal evolution
Source: Nat Commun. 2025 Jun 5;16:4648. doi: 10.1038/s41467-025-59974-x (PMC12141501; doi:10.1038/s41467-025-59974-x)
Supplement: Supplementary file 1 — Supplementary information [file 41467_2025_59974_MOESM1_ESM.pdf]

Supplementary Information

Two major ecological shifts shaped 60 million years of ungulate faunal evolution

Fernando Blanco [a,b,c*] , Ignacio A. Lazagabaster [c, d, e], Óscar Sanisidro [f] , Faysal Bibi [c], Nicola S. Heckeberg [g,h], María Ríos [i], Bastien Mennecart [j] , María Teresa Alberdi [k], Jose Luis Prado [l], Juha Saarinen [m], Daniele Silvestro [a,b,n], Johannes Müller [c], Joaquín Calatayud [o] & Juan L. Cantalapiedra [k, c, f]

*Corresponding author: Fernando Blanco
Email: fblancosegovia@gmail.com

This PDF file includes:

Supplementary Figures 1 to 22
Supplementary Tables 1 to 2

Supporting Figures

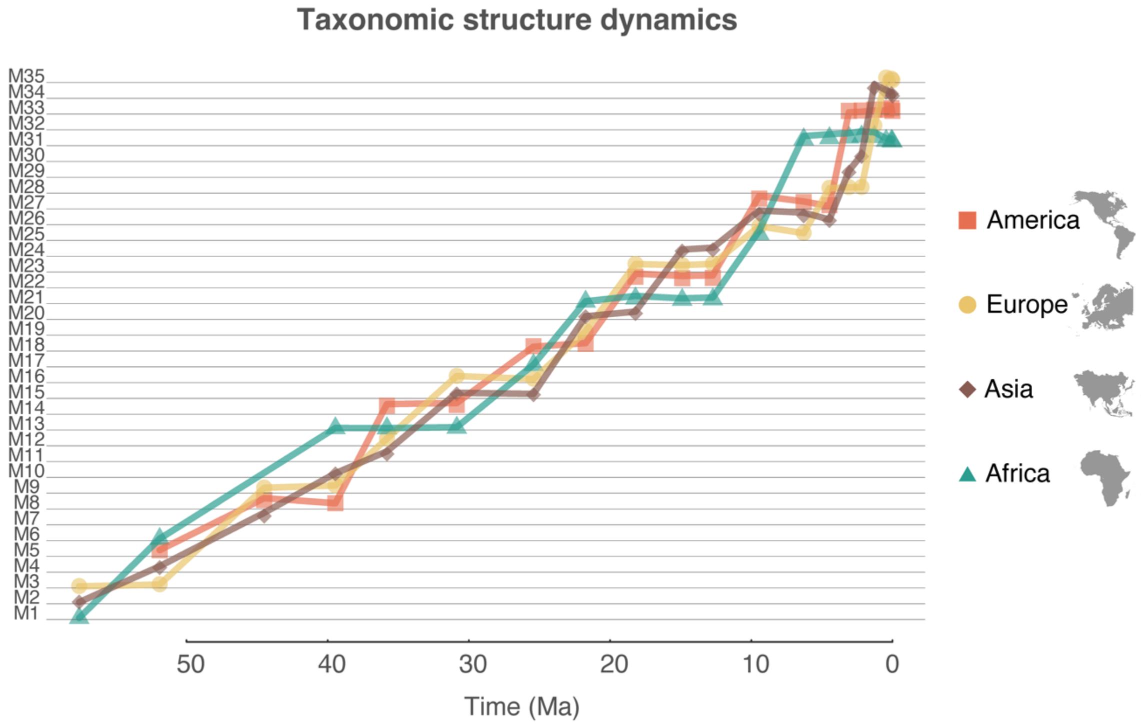

Supplementary Figure 1. Taxonomic structure dynamics of large herbivores. Succession of taxonomic modules (M1 to M35) derived from network analysis, plotted against time (in million years, Ma). Each module represents a distinct taxonomic structure based on species and continent-stage (nodes in the network) associations. Different colors and shapes indicate continent-stages across the different continents (America, Europe, Asia, and Africa; see legend). Lines represent module transitions within each continent, illustrating large-scale patterns of taxonomic succession. Map outlines are from freevectormaps.com.

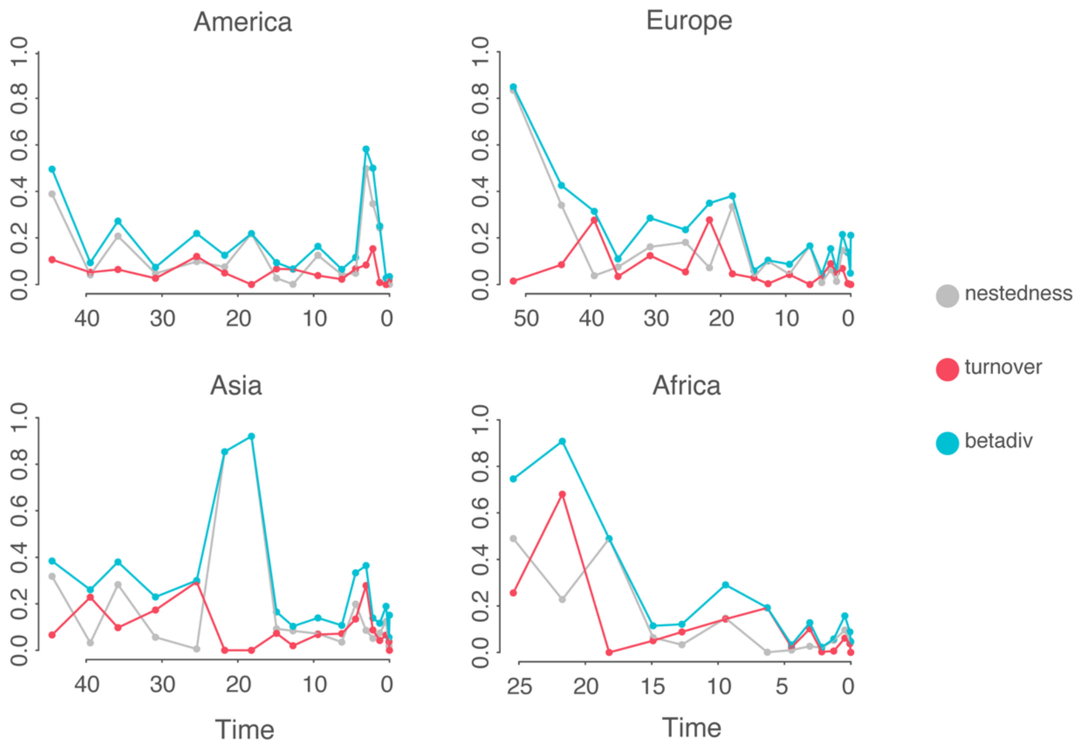

Supplementary Figure 2. Beta diversity evolution over the Cenozoic. Evolution of the beta diversity and its components (nestedness and turnover) are plotted by colors against time in Myrs for each continent.

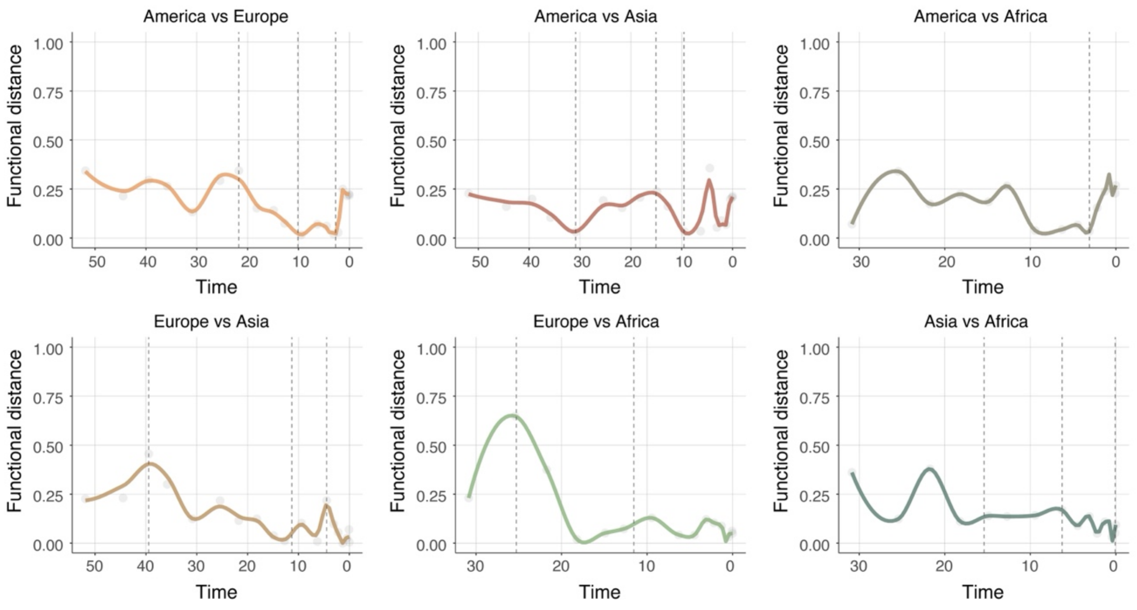

Supplementary Figure 3. Continents' functional distance through time. Functional distance between continent pairs calculated from the functional space in Fig. 1B is plotted against time in myr**.** Line colors are derived from the combination of continent color in Fig. 1A. Dotted lines are inflection points from regression models with segmented relationships (see methods).

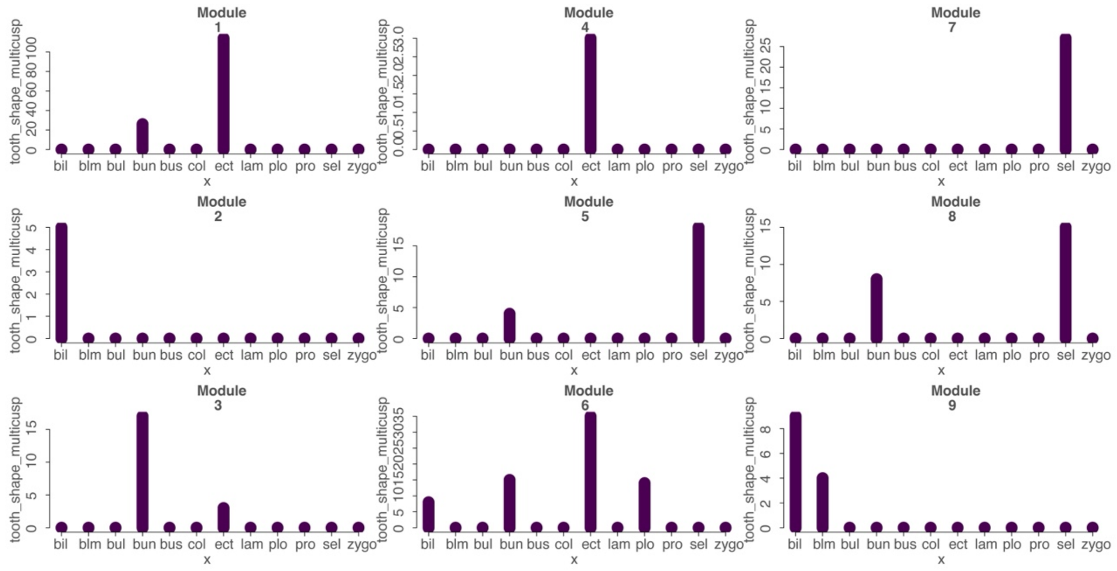

Supplementary Figure 4. Most abundant trait states by module. Traits state abundance are calculated for the traits of significant higher IndVal values (see methods) in each module. X axis represents the different states corresponding to the trait.

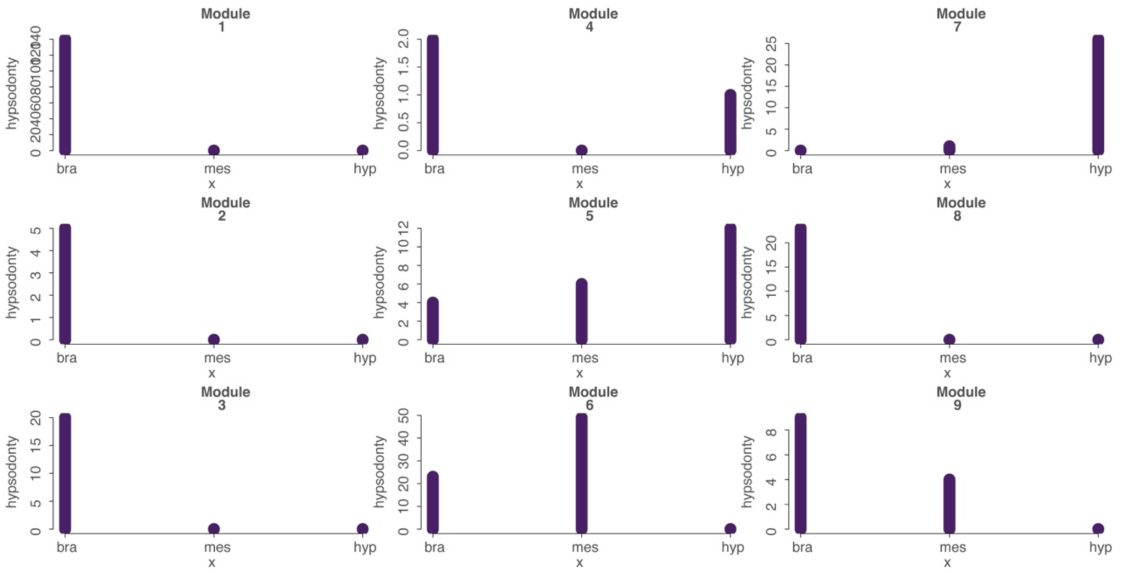

Supplementary Figure 5. Most abundant trait states by module. Traits state abundance are calculated for the traits of significant higher IndVal values (see methods) in each module. X axis represents the different states corresponding to the trait.

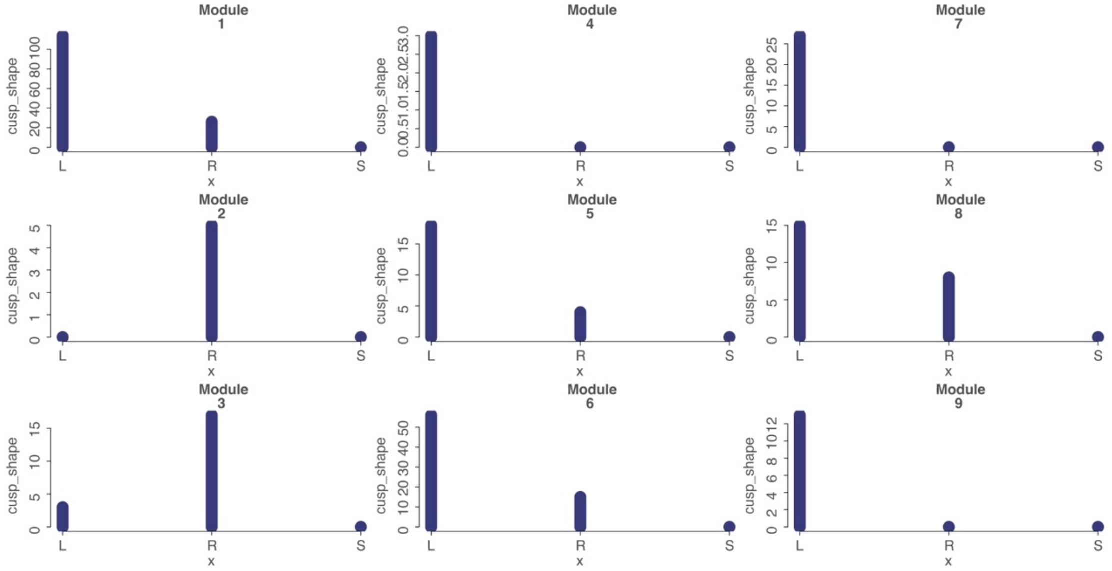

Supplementary Figure 6. Most abundant trait states by module. Traits state abundance are calculated for the traits of significant higher IndVal values (see methods) in each module. X axis represents the different states corresponding to the trait.

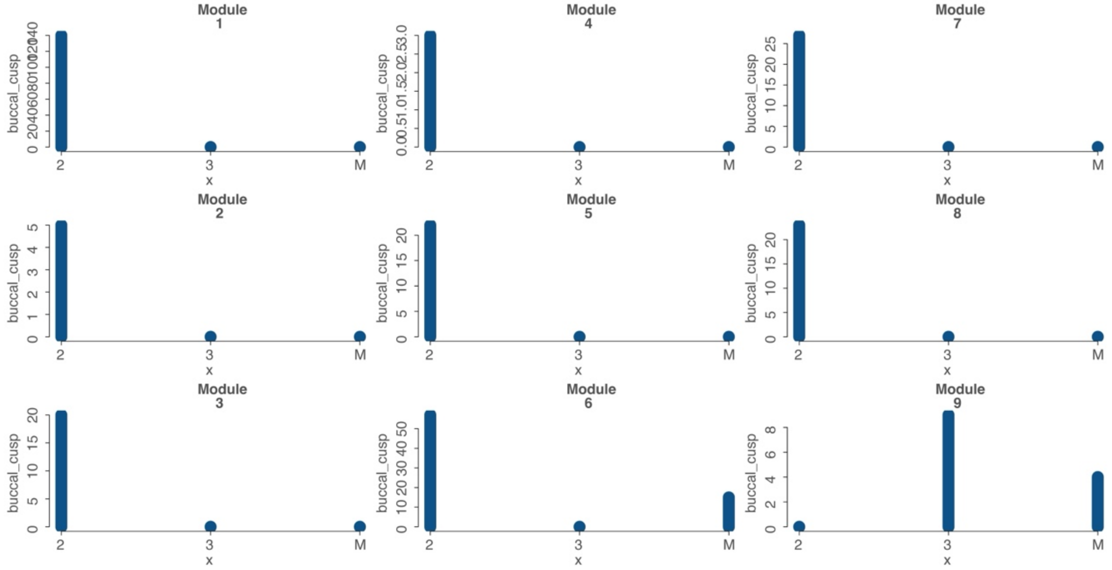

Supplementary Figure 7. Most abundant trait states by module. Traits state abundance are calculated for the traits of significant higher IndVal values (see methods) in each module. X axis represents the different states corresponding to the trait.

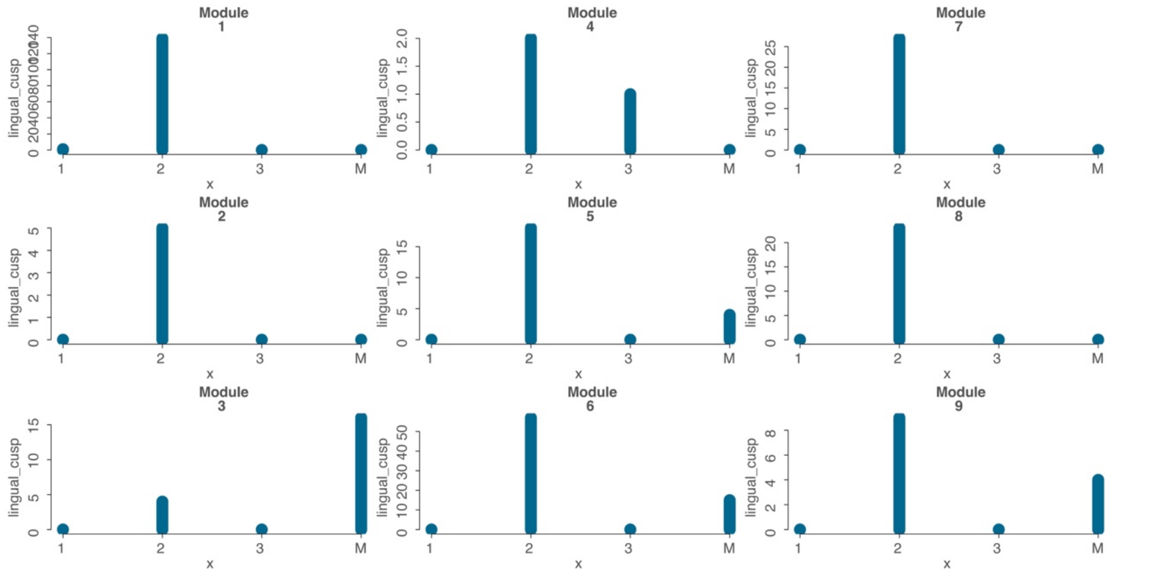

Supplementary Figure 8. Most abundant trait states by module. Traits state abundance are calculated for the traits of significant higher IndVal values (see methods) in each module. X axis represents the different states corresponding to the trait.

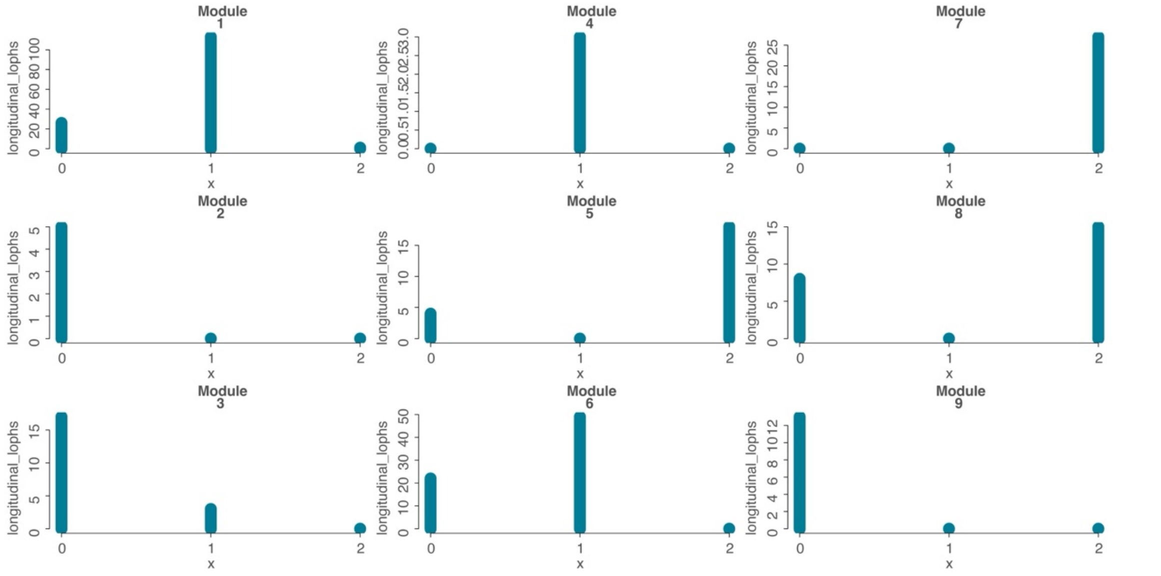

Supplementary Figure 9. Most abundant trait states by module. Traits state abundance are calculated for the traits of significant higher IndVal values (see methods) in each module. X axis represents the different states corresponding to the trait.

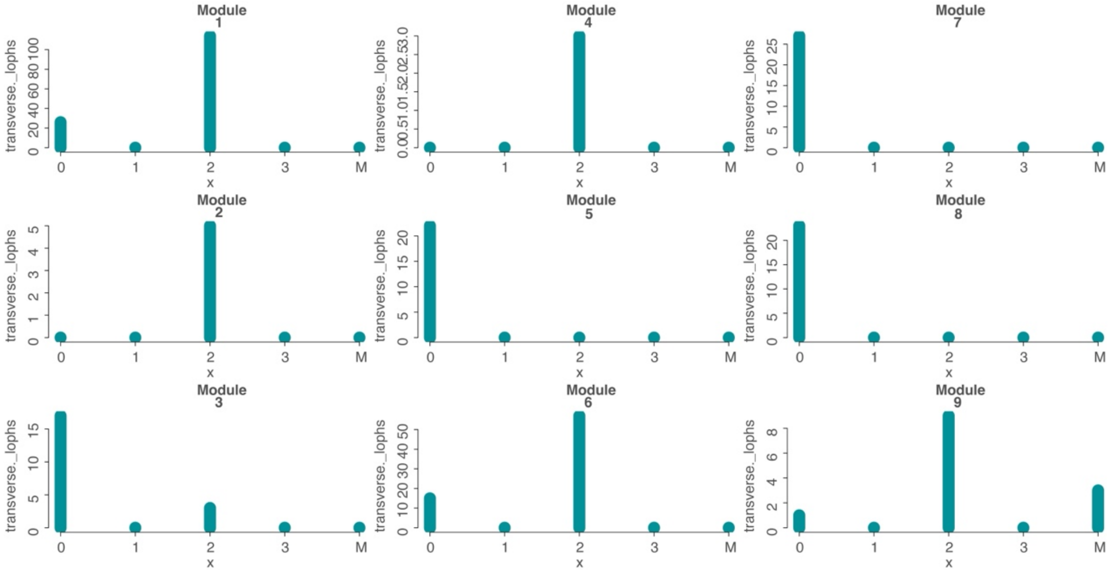

Supplementary Figure 10. Most abundant trait states by module. Traits state abundance are calculated for the traits of significant higher IndVal values (see methods) in each module. X axis represents the different states corresponding to the trait.

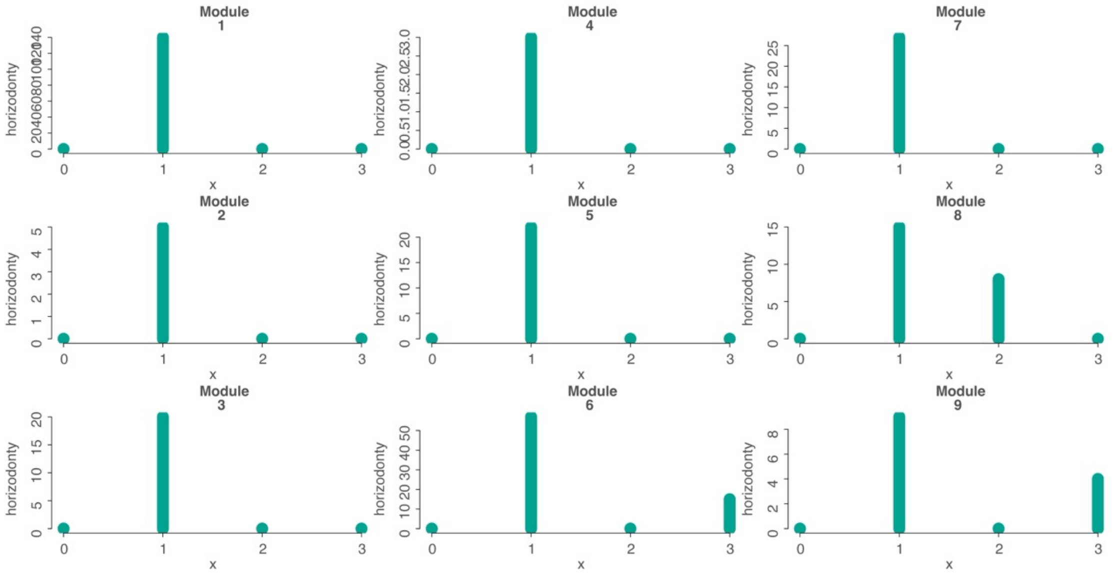

Supplementary Figure 11. Most abundant trait states by module. Traits state abundance are calculated for the traits of significant higher IndVal values (see methods) in each module. X axis represents the different states corresponding to the trait.

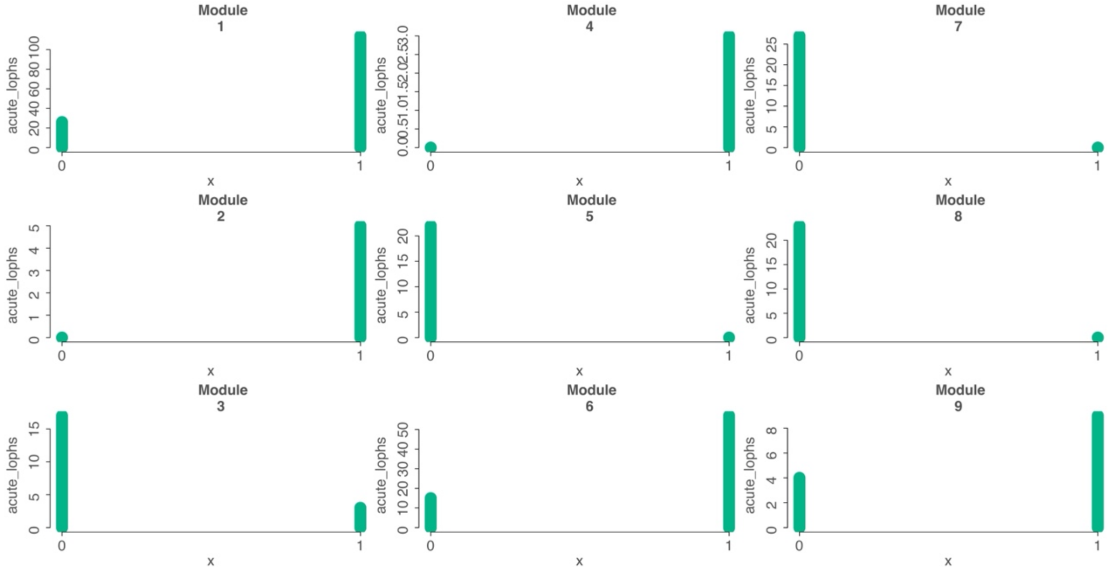

Supplementary Figure 12. Most abundant trait states by module. Traits state abundance are calculated for the traits of significant higher IndVal values (see methods) in each module. X axis represents the different states corresponding to the trait.

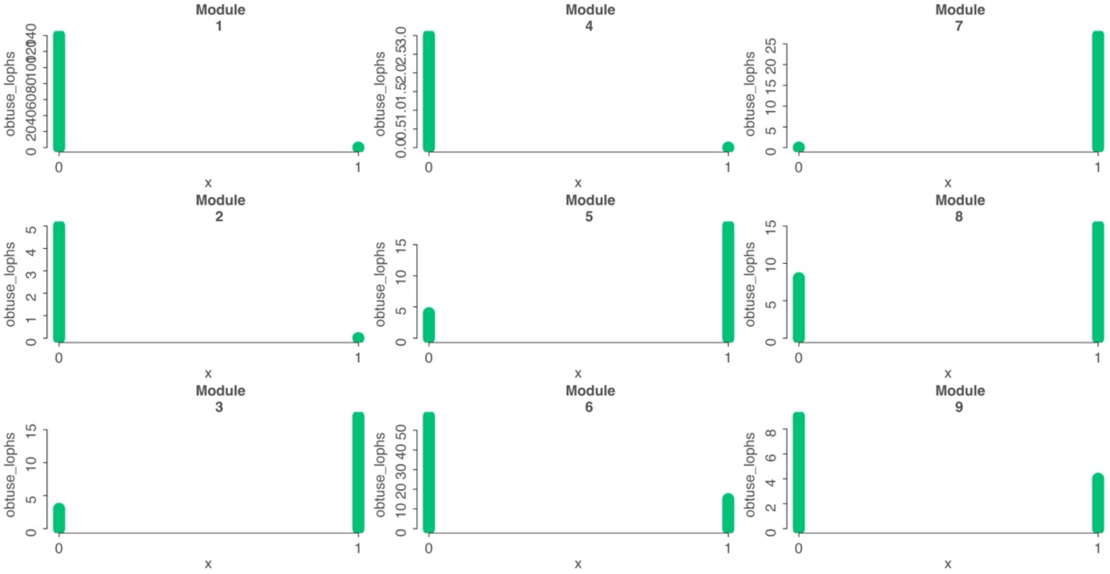

Supplementary Figure 13. Most abundant trait states by module. Traits state abundance are calculated for the traits of significant higher IndVal values (see methods) in each module. X axis represents the different states corresponding to the trait.

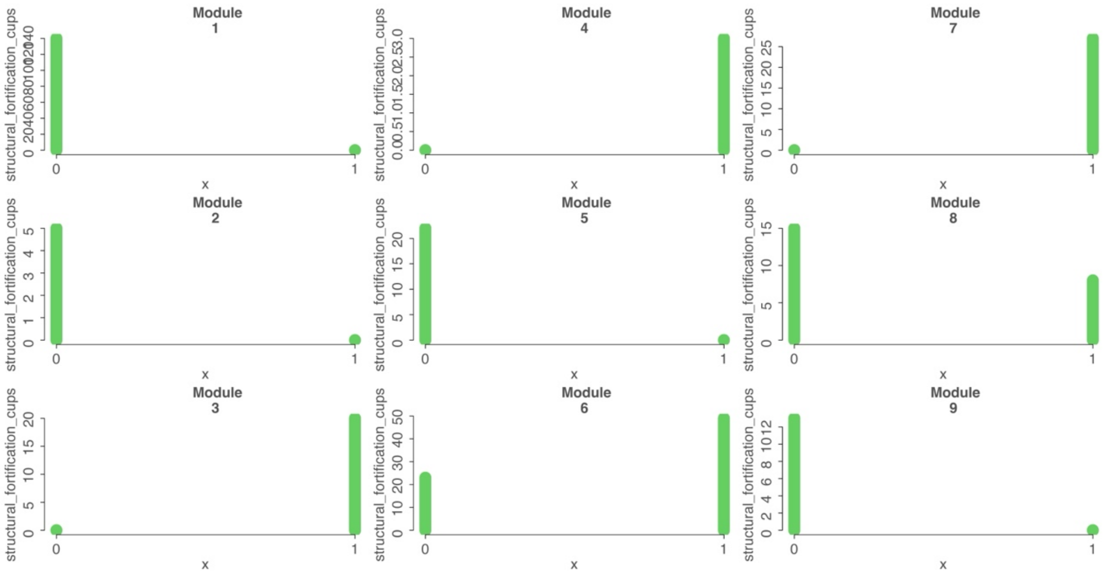

Supplementary Figure 14. Most abundant trait states by module. Traits state abundance are calculated for the traits of significant higher IndVal values (see methods) in each module. X axis represents the different states corresponding to the trait.

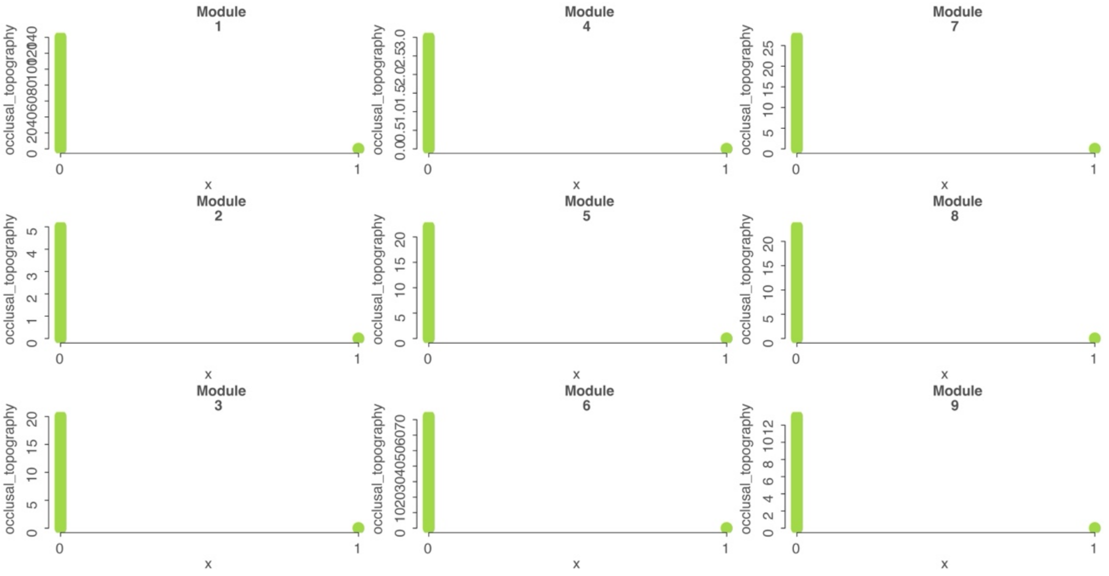

Supplementary Figure 15. Most abundant trait states by module. Traits state abundance are calculated for the traits of significant higher IndVal values (see methods) in each module. X axis represents the different states corresponding to the trait.

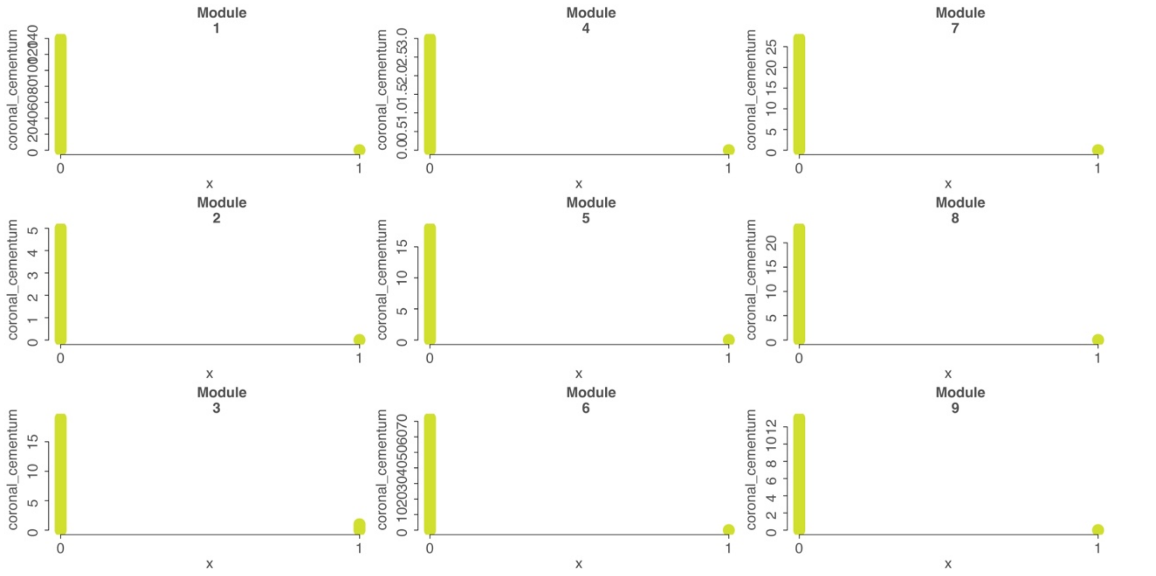

Supplementary Figure 16. Most abundant trait states by module. Traits state abundance are calculated for the traits of significant higher IndVal values (see methods) in each module. X axis represents the different states corresponding to the trait.

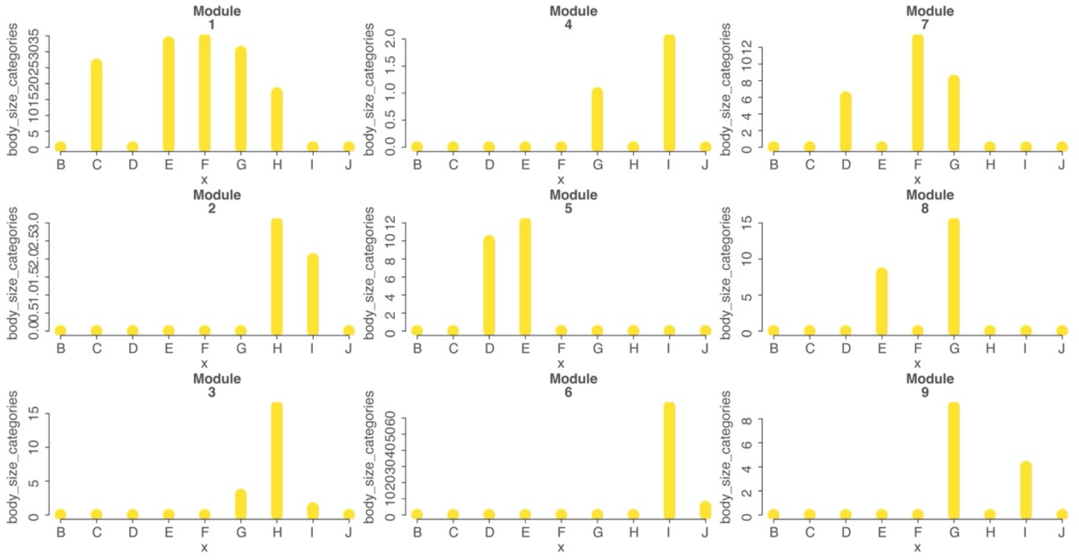

Supplementary Figure 17. Most abundant trait states by module. Traits state abundance are calculated for the traits of significant higher IndVal values (see methods) in each module. X axis represents the different states corresponding to the trait.

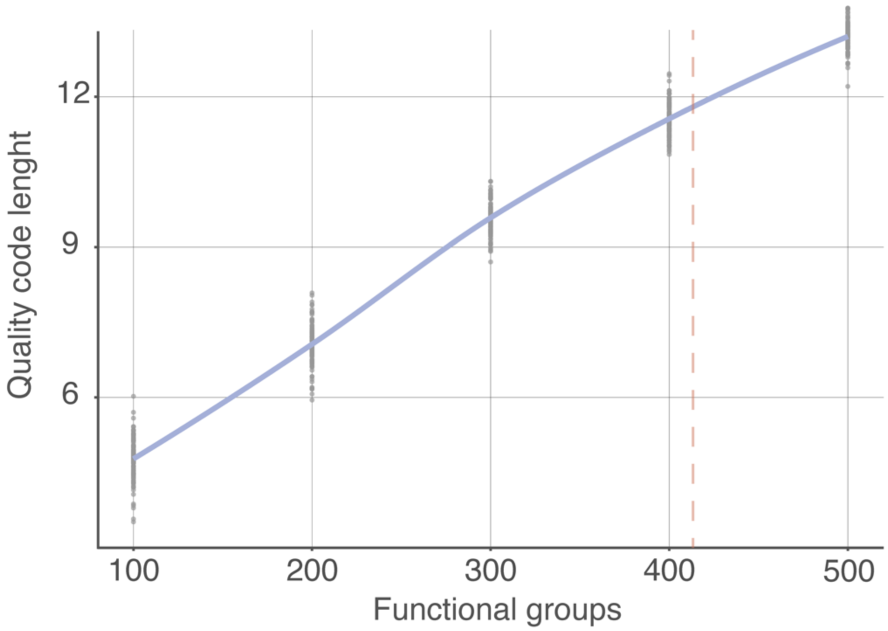

Supplementary Figure 18. K-means sensitivity analysis. Quality code length (measure of modularity) is plotted against the number of modules obtained from the network analysis for all k-means randomization (see methods). Dashed red line represents the number of modules used for the analysis (405).

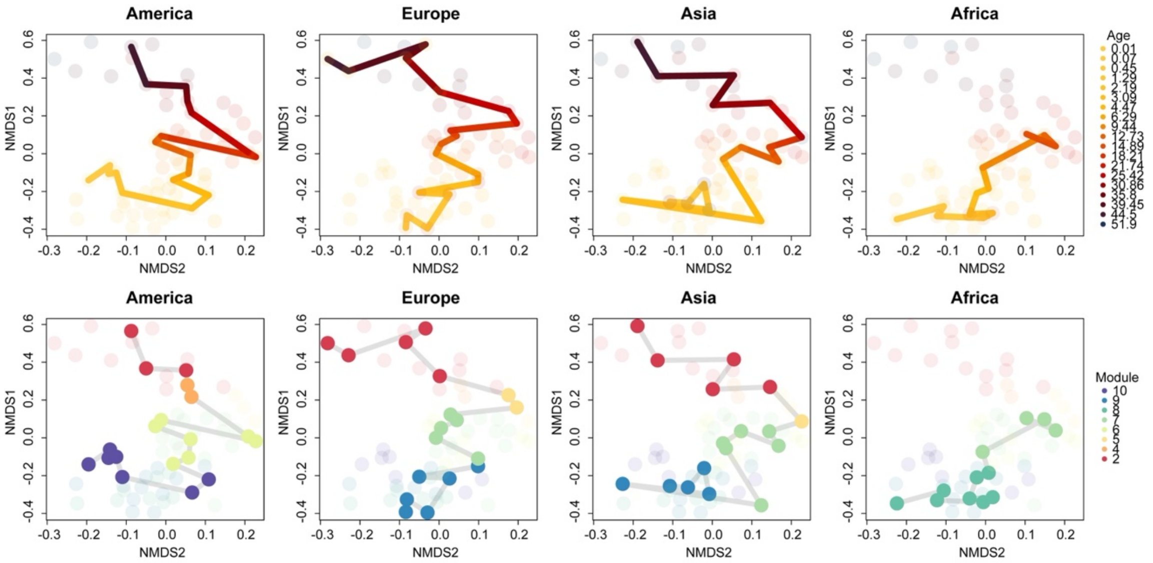

Supplementary Figure 19. Randomized results of functional structure. Functional space for each continent from time bin functional distances calculated as turnover plotted in two dimensions through NMDS (see methods). Dots represent functional time bins in each continent. Above color-scale indicates time in Myrs. Colors below represent the different modules from the network analysis (Fig. 1A).

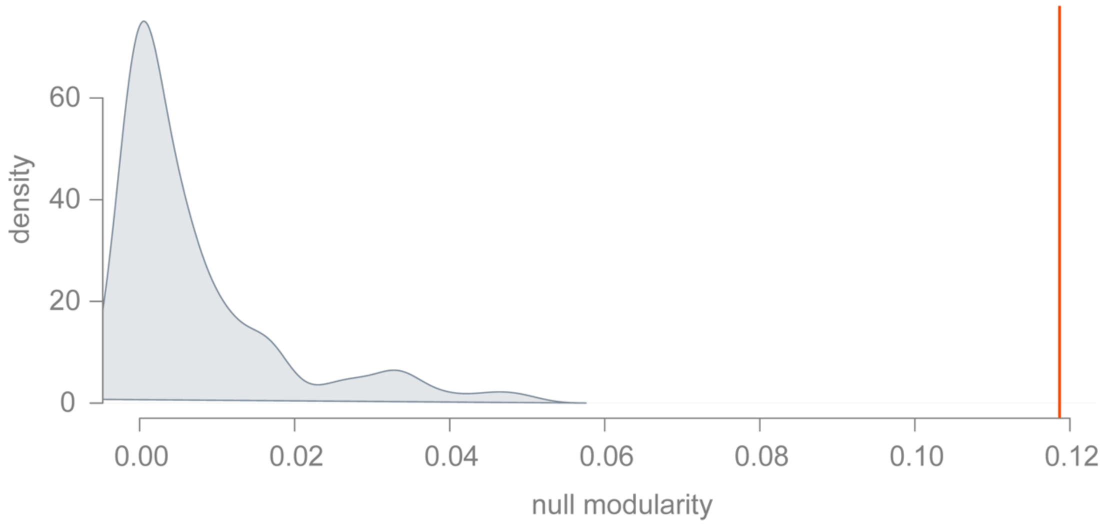

Supplementary Figure 20. Observed and simulated modularity of functional networks. Gray density diagrams show the modularity scores of 100 random networks compared with the observed network (red vertical line).

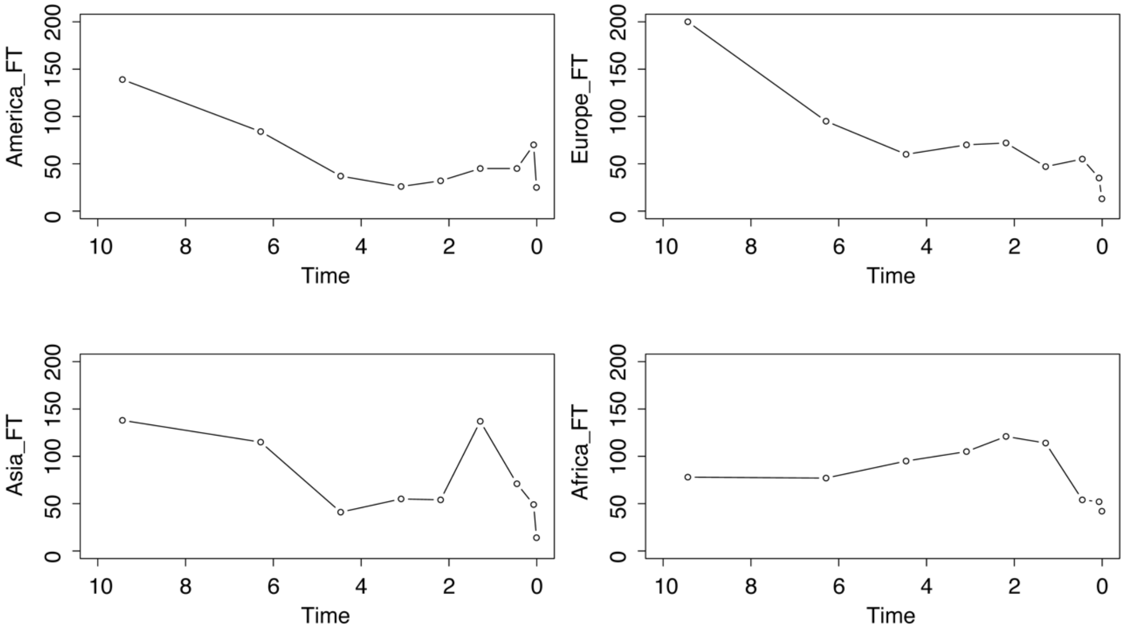

Supplementary Figure 21. Functional type richness over the last 10 Ma. The total number of functional types in each continent is plotted against time for the last 10Ma.

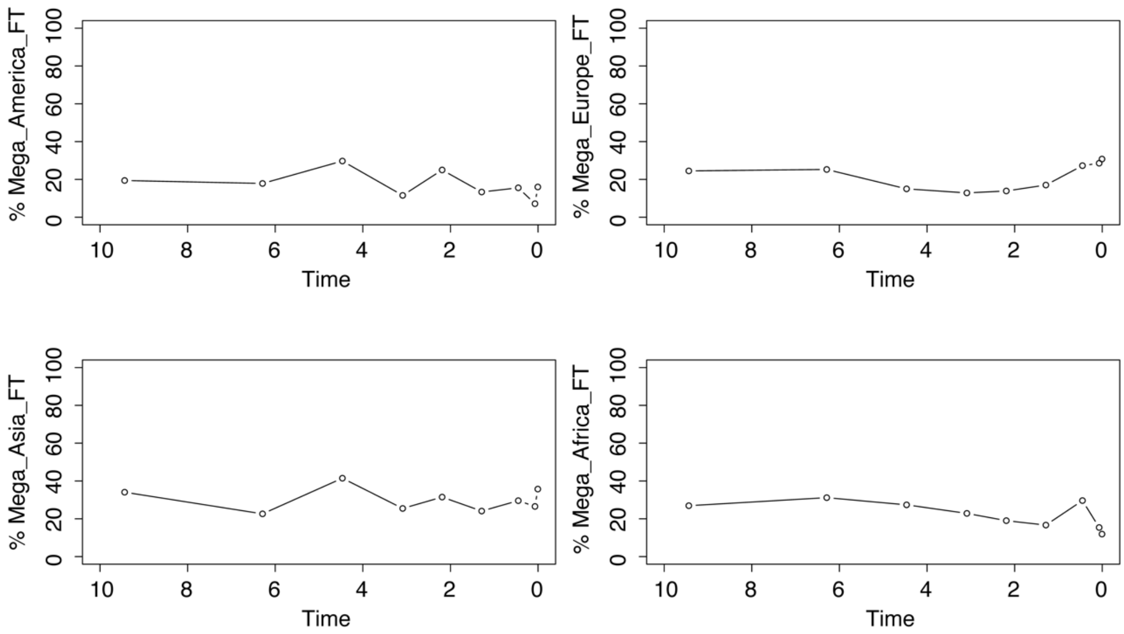

Supplementary Figure 22. Percentage of megaherbivores functional types over the last 10 Ma. Percentage of the total functional types belonging to megaherbivore in each continent is plotted against time for the last 10Ma.

Supplementary Table 1. Probability of obtaining the same module partition with similarity >0.5 and >0.75 (Robustness) for the functional network.

Module	Robustness >0.5	Robustness >0.75
1	1	1
2	1	1
3	1	1
4	1	1
5	1	1
6	1	1
7	1	1
8	1	1
9	1	1

Supplementary Table 2. Linear model comparison for functional diversity using occurrences*continent and occurrences*occurrences^2. K= number of estimated parameters in the model, AICc= Akaike information criterion , LL= log-likelihood.

M names	*K*	*AICc*	*Delta AICc*	*LL*
occurrences*continent	9	14.66	0.00	3.38
occurrences*occurrences^2.	5	39.68	25.22	-14.34
