# Supplementary material for: Two major ecological shifts shaped 60 million years of ungulate faunal evolution
Source: Nat Commun. 2025 Jun 5;16:4648. doi: 10.1038/s41467-025-59974-x (PMC12141501; doi:10.1038/s41467-025-59974-x)
Supplement: Supplementary file 2 — Description of addtional supplementary file [file 41467_2025_59974_MOESM2_ESM.pdf]

Description of Additional Supplementary Files

Supplementary Data 1. Fidelity, Affinity and IndVal values for the network nodes in each module.

Supplementary Code 1. All codes are used to perform the analyses and generate the figures in Blanco et al. 2025.
