# Supplementary material for: Two major ecological shifts shaped 60 million years of ungulate faunal evolution
Source: Nat Commun. 2025 Jun 5;16:4648. doi: 10.1038/s41467-025-59974-x (PMC12141501; doi:10.1038/s41467-025-59974-x)
Supplement: Supplementary file 5 — Reporting Summary [file 41467_2025_59974_MOESM5_ESM.pdf]

nature portfolio

Corresponding author(s): Fernando Blanco

Last updated by author(s): Apr 15, 2025

Reporting Summary

Nature Portfolio wishes to improve the reproducibility of the work that we publish. This form provides structure for consistency and transparency in reporting. For further information on Nature Portfolio policies, see our Editorial Policies and the Editorial Policy Checklist.

Statistics

For all statistical analyses, confirm that the following items are present in the figure legend, table legend, main text, or Methods section.

n/a	Confirmed	
☐	☒	The exact sample size (*n*) for each experimental group/condition, given as a discrete number and unit of measurement
☒	☐	A statement on whether measurements were taken from distinct samples or whether the same sample was measured repeatedly
☐	☒	The statistical test(s) used AND whether they are one- or two-sided<br>*Only common tests should be described solely by name; describe more complex techniques in the Methods section.*
☐	☒	A description of all covariates tested
☐	☒	A description of any assumptions or corrections, such as tests of normality and adjustment for multiple comparisons
☐	☒	A full description of the statistical parameters including central tendency (e.g. means) or other basic estimates (e.g. regression coefficient) AND variation (e.g. standard deviation) or associated estimates of uncertainty (e.g. confidence intervals)
☐	☒	For null hypothesis testing, the test statistic (e.g. *F*, *t*, *r*) with confidence intervals, effect sizes, degrees of freedom and *P* value noted<br>*Give P values as exact values whenever suitable.*
☒	☐	For Bayesian analysis, information on the choice of priors and Markov chain Monte Carlo settings
☐	☒	For hierarchical and complex designs, identification of the appropriate level for tests and full reporting of outcomes
☐	☒	Estimates of effect sizes (e.g. Cohen's *d*, Pearson's *r*), indicating how they were calculated

Our web collection on statistics for biologists contains articles on many of the points above.

Software and code

Policy information about availability of computer code

Data collection	The data were collected from PBDB (paleobiodb.org) and NOW databases (https://nowdatabase.org) and selected literature.
Data analysis	All the analysis were performed in the open software R (www.R-project.org).

For manuscripts utilizing custom algorithms or software that are central to the research but not yet described in published literature, software must be made available to editors and reviewers. We strongly encourage code deposition in a community repository (e.g. GitHub). See the Nature Portfolio guidelines for submitting code & software for further information.

Data

Policy information about availability of data

All manuscripts must include a data availability statement. This statement should provide the following information, where applicable:

- Accession codes, unique identifiers, or web links for publicly available datasets
- A description of any restrictions on data availability
- For clinical datasets or third party data, please ensure that the statement adheres to our policy

Source data for all analyses and figures are provided in the accompanying Source Data file. All data used in this study are also available at :https://github.com/f-blanco/ecoherb and https://figshare.com/authors/Fernando_Blanco/9731162

Research involving human participants, their data, or biological material

Policy information about studies with human participants or human data. See also policy information about sex, gender (identity/presentation), and sexual orientation and race, ethnicity and racism.

Reporting on sex and gender	*Use the terms sex (biological attribute) and gender (shaped by social and cultural circumstances) carefully in order to avoid confusing both terms. Indicate if findings apply to only one sex or gender; describe whether sex and gender were considered in study design; whether sex and/or gender was determined based on self-reporting or assigned and methods used. Provide in the source data disaggregated sex and gender data, where this information has been collected, and if consent has been obtained for sharing of individual-level data; provide overall numbers in this Reporting Summary. Please state if this information has not been collected. Report sex- and gender-based analyses where performed, justify reasons for lack of sex- and gender-based analysis.*
Reporting on race, ethnicity, or other socially relevant groupings	*Please specify the socially constructed or socially relevant categorization variable(s) used in your manuscript and explain why they were used. Please note that such variables should not be used as proxies for other socially constructed/relevant variables (for example, race or ethnicity should not be used as a proxy for socioeconomic status). Provide clear definitions of the relevant terms used, how they were provided (by the participants/respondents, the researchers, or third parties), and the method(s) used to classify people into the different categories (e.g. self-report, census or administrative data, social media data, etc.) Please provide details about how you controlled for confounding variables in your analyses.*
Population characteristics	*Describe the covariate-relevant population characteristics of the human research participants (e.g. age, genotypic information, past and current diagnosis and treatment categories). If you filled out the behavioural & social sciences study design questions and have nothing to add here, write "See above."*
Recruitment	*Describe how participants were recruited. Outline any potential self-selection bias or other biases that may be present and how these are likely to impact results.*
Ethics oversight	*Identify the organization(s) that approved the study protocol.*

Note that full information on the approval of the study protocol must also be provided in the manuscript.

Field-specific reporting

Please select the one below that is the best fit for your research. If you are not sure, read the appropriate sections before making your selection.

☐ Life sciences ☐ Behavioural & social sciences ☒ Ecological, evolutionary & environmental sciences

For a reference copy of the document with all sections, see nature.com/documents/nr-reporting-summary-flat.pdf

Ecological, evolutionary & environmental sciences study design

All studies must disclose on these points even when the disclosure is negative.

Study description	We characterize the functional structure of global large herbivores during the last 60 million years. For that, we used a combination of network analysis, functional space and functional diversity analysis. We found that ungulate continental assemblages exhibited prolonged ecological stability interrupted by irreversible reorganizations associated with abiotic events. During the early Cenozoic, continental assemblages were dominated by mid-sized browsers with low-crowned teeth, which showed increasing functional diversity. Around 21 Ma, the formation of a land bridge between Eurasia and Africa triggered a first major global transition towards a new functional system featuring a prevalence of large browsers with mid- to high-crowned molars. Functional diversity continued to increase, peaking around 10 Ma. Shortly after, aridification and the spread of C4-dominated vegetation rendered a second tipping point towards a fauna characterized by grazers and browsers with high and low crowned teeth. A global decline in ungulate functional diversity began 10 Ma ago and accelerated around 2.5 Ma, yet the functional structure of these faunas remained stable in the latest Cenozoic. Large mammal evolutionary history reflects two key transitions, aligning with major tectonic and climatic events.
Research sample	We collected information for all the large herbivores in the orders Artiodactyla, Perissodactyla and Proboscidea worldwide expaning the last 60 million years. The complete database encompases 22,028 occurrences of 3,046 species distributed in 10,680 sites worldwide. The data also includes 14 functional traits for each species.
Sampling strategy	A detailed sampling analysis is described in the Methods section.
Data collection	The data was collected from PBDB (paleobiodb.org) and NOW databases (https://nowdatabase.org) and selected literature.
Timing and spatial scale	The data include all the occurences in the orders Artiodactyla, Perissodactyla and Proboscidea worldwide expaning the last 60 million years.
Data exclusions	No data were excluded from the analysis.
Reproducibility	All attemps to repeat the analysis were sucessfull. Source data for all analyses and figures are provided in the accompanying Source

Reproducibility	Data file. All data used in this study are also available at :https://github.com/f-blanco/ecoherb and https://figshare.com/authors/Fernando_Blanco/9731162
Randomization	A detailed sampling analysis is described in the Methods section and Supplementary Text, including randomization, null models, etc.
Blinding	Not applicable

Did the study involve field work? ☐ Yes ☒ No

Reporting for specific materials, systems and methods

We require information from authors about some types of materials, experimental systems and methods used in many studies. Here, indicate whether each material, system or method listed is relevant to your study. If you are not sure if a list item applies to your research, read the appropriate section before selecting a response.

Materials & experimental systems

n/a	Involved in the study
☒	☐ Antibodies
☒	☐ Eukaryotic cell lines
☒	☐ Palaeontology and archaeology
☒	☐ Animals and other organisms
☒	☐ Clinical data
☒	☐ Dual use research of concern
☒	☐ Plants

Methods

n/a	Involved in the study
☒	☐ ChIP-seq
☒	☐ Flow cytometry
☒	☐ MRI-based neuroimaging

Plants

Seed stocks	*Report on the source of all seed stocks or other plant material used. If applicable, state the seed stock centre and catalogue number. If plant specimens were collected from the field, describe the collection location, date and sampling procedures.*
Novel plant genotypes	*Describe the methods by which all novel plant genotypes were produced. This includes those generated by transgenic approaches, gene editing, chemical/radiation-based mutagenesis and hybridization. For transgenic lines, describe the transformation method, the number of independent lines analyzed and the generation upon which experiments were performed. For gene-edited lines, describe the editor used, the endogenous sequence targeted for editing, the targeting guide RNA sequence (if applicable) and how the editor was applied.*
Authentication	*Describe any authentication procedures for each seed stock used or novel genotype generated. Describe any experiments used to assess the effect of a mutation and, where applicable, how potential secondary effects (e.g. second site T-DNA insertions, mosiacism, off-target gene editing) were examined.*
